# Human Omental-Derived Adipose Stem Cells Increase Ovarian Cancer Proliferation, Migration, and Chemoresistance

**DOI:** 10.1371/journal.pone.0081859

**Published:** 2013-12-02

**Authors:** Aleksandra Nowicka, Frank C. Marini, Travis N. Solley, Paula B. Elizondo, Yan Zhang, Hadley J. Sharp, Russell Broaddus, Mikhail Kolonin, Samuel C. Mok, Melissa S. Thompson, Wendy A. Woodward, Karen Lu, Bahar Salimian, Deepak Nagrath, Ann H. Klopp

**Affiliations:** 1 Department of Radiation Oncology, The University of Texas MD Anderson Cancer Center, Houston, Texas, United States of America; 2 Department of Cancer Biology, Wake Forest Baptist Medical Center, Winston-Salem, North Carolina, United States of America; 3 Asuragen Inc., Austin, Texas, United States of America; 4 Department of Pathology Administration, The University of Texas MD Anderson Cancer Center, Houston, Texas, United States of America; 5 Center for Stem Cell and Regenerative Medicine, The University of Texas Health Science Center at Houston, Houston, Texas, United States of America; 6 Department of Gynecologic Oncology and Reproductive Medicine, The University of Texas MD Anderson Cancer Center, Houston, Texas, United States of America; 7 Gulf Coast Consortia, Houston, Texas, United States of America; 8 Chemical and Biomolecular Engineering Department, Rice University, Houston, Texas, United States of America; Rutgers - New Jersey Medical School, United States of America

## Abstract

**Objectives:**

Adipose tissue contains a population of multipotent adipose stem cells (ASCs) that form tumor stroma and can promote tumor progression. Given the high rate of ovarian cancer metastasis to the omental adipose, we hypothesized that omental-derived ASC may contribute to ovarian cancer growth and dissemination.

**Materials and Methods:**

We isolated ASCs from the omentum of three patients with ovarian cancer, with (O-ASC4, O-ASC5) and without (O-ASC1) omental metastasis. BM-MSCs, SQ-ASCs, O-ASCs were characterized with gene expression arrays and metabolic analysis. Stromal cells effects on ovarian cancer cells proliferation, chemoresistance and radiation resistance was evaluated using co-culture assays with luciferase-labeled human ovarian cancer cell lines. Transwell migration assays were performed with conditioned media from O-ASCs and control cell lines. SKOV3 cells were intraperitionally injected with or without O-ASC1 to track *in-vivo* engraftment.

**Results:**

O-ASCs significantly promoted *in* *vitro* proliferation, migration chemotherapy and radiation response of ovarian cancer cell lines. O-ASC4 had more marked effects on migration and chemotherapy response on OVCA 429 and OVCA 433 cells than O-ASC1. Analysis of microarray data revealed that O-ASC4 and O-ASC5 have similar gene expression profiles, in contrast to O-ASC1, which was more similar to BM-MSCs and subcutaneous ASCs in hierarchical clustering. Human O-ASCs were detected in the stroma of human ovarian cancer murine xenografts but not uninvolved ovaries.

**Conclusions:**

ASCs derived from the human omentum can promote ovarian cancer proliferation, migration, chemoresistance and radiation resistance *in-vitro*. Furthermore, clinical O-ASCs isolates demonstrate heterogenous effects on ovarian cancer *in-vitro*.

## Introduction

Ovarian cancer is the most lethal gynecologic malignancy, resulting in 16,000 deaths per year in the United States [1]. The mortality and morbidity of ovarian cancer is due to a high rate of intraperitoneal dissemination and the development of chemotherapy-resistant tumors despite initially chemoresponsive disease. 

Intraperitoneal dissemination of ovarian cancer frequently results in the formation of metastasis in the omentum, the well-vascularized and innervated fatty tissue that lies over the bowel. The reason why the omentum is the preferable “soil” for metastatic ovarian cancer cells remains unknown. The response of ovarian cancer metastasis in the omentum to chemotherapy is an independent predictor of death from ovarian cancer, suggesting that interactions between ovarian cancer and the omental microenvironment are an important driver of clinical outcome [2].

We hypothesized that the omentum is a hospitable environment for the formation of ovarian cancer metastasis due to a population of tumor tropic mesenchymal stem cells in the omental adipose. Recently, we characterized a population of multi-potent adipose-derived mesenchymal stem cells from the omentum (O-ASCs) of two patients with omental disease and one without. Each isolate was confirmed to possess multi-potent differentiation potential as expected from MSC. ASCs resemble bone marrow-derived mesenchymal stem cells (BM-MSCs) which have the capacity to migrate into tumors and can have been shown to promote tumor initiation, growth, vascularization, metastasis, and resistance to chemotherapy in many tumor models [3-6]. We investigated the effects of O-ASCs from patients with and without omental metastasis on ovarian cancer cell lines.

## Materials and Methods

### Cell lines

The human ovarian carcinoma cell lines OVCA 429, OVCA 433, and A2780 were obtained from Dr. Samuel C. Mok (The University of Texas MD Anderson Center, Houston, TX). OVCA 429 and OVCA 433 cell lines were established cell lines derived from patients with late-stage serous ovarian adenocarcinomas, as described by Bast et al [7]. The human ovarian carcinoma cell lines SKOV3 and A2780 and human BM-MSCs were obtained from American Type Culture Collection (Manassas, VA). All cell lines were maintained in DMEM (Mediatech, Inc., Manassas, VA) containing 10% fetal bovine serum (FBS) (HyClone, Logan, UT) and 1% penicillin and streptomycin (Mediatech, Manassas, VA) at 37°C in a humidified atmosphere of 5% CO2. 

### Human O-ASCs and SQ-ASCs isolation

Under The MD Anderson Institutional Review Board-approved protocol, grossly normal-appearing human omentum and subcutaneous adipose tissue were obtained during the staging procedures of patients with ovarian cancer. Informed consent for tissue banking was obtained from each patient. All clinical investigation has been conducted through the principals expressed in the Declaration of Helinski. Written consent was obtained from each patient. O-ASCs and SQ-ASCs were isolated according to previously published protocols [8]. O-ASC1 was isolated from a patient (body mass index (BMI) = 25.2 kg/m^2^) with recurrent endometriod adenocarcinoma of the endometrium and ovary without omental metastasis. O-ASC4 and O-ASC5 were isolated from from patients with high-grade serous ovarian cancer with omental involvement. The BMI for patients from whom O-ASC4 and 5 were derived were 32.4 kg/m^2^ and 22 kg/m^2^, respectively. O-ASC5 was used for gene expression array and cell surface marker expression but was lost after serial passaging *in-vitro* and thus couldn’t used in functional assays. The isolated O-ASCs and SQ-ASCs were cultured in α-MEM medium (Mediatech, Manassas, VA) with 20% FBS (HyClone, Logan, UT) and 1% penicillin streptomycin, and L-glutamine (Mediatech, Manassas, VA). 

### O-ASCs line characterization

After isolation, the cells were expanded *in-vitro* in α-MEM medium (Mediatech, Manassas, VA). Cell surface marker expression was characterized by flow cytometry analysis. O-ASCs were characterized at early passage (maximum 5) using antibodies specific for the following markers: CD11b, CD29, CD34, CD44, CD45, CD 90, EpCam (Becton Dickinson, Franklin Lakes, NJ), and CD105 (BioLegend, San Diego, CA). 

To confirm the adipogenic potential of O-ASCs and BM-MSCs, we incubated 10^5^ cells in adipogenic induction media (DMEM medium with 10% FBS, 45 g/L glucose, L-glutamine, 1% penicillin and streptomycin, 10 µg/ml insulin, 500 µM 3-isobutyl-1-methylxanthine, 1 µM dexamethasone, and 200 µM indomethacin). After 72 hours, maintenance medium (DMEM Mediatech, Manassas, VA) with 10% FBS, 45 g/L glucose, L-glutamine, 10 µg/ml insulin, 1% penicillin and streptomycin) was added to the cells. The maintenance medium was changed 2 times per week during 10 days of incubation. At the indicated times, we performed oil red O (Sigma–Aldrich, St. Louis, MO) histochemical staining of the cytoplasmic inclusions of neutral lipids of functional adipocytes.

Osteoblastic differentiation O-ASCs and BM-MSCs was performed using 5 x10^4^ cells. After 3 weeks incubation in osteoblast induction medium (NH OsteoDiff medium, Bergisch Gladbach, Miltenyi Biotec GmbH, Germany), extracellular calcium deposits were stained using Alizarin Red S (Sigma–Aldrich, St. Louis, MO). 

We confirmed the cells chondrogenic potential using 10^6^ cells, which were incubated in 2 ml of chondrogenic medium (DMEM medium with 45 g/L glucose, L-glutamine, 1% penicillin and streptomycin, 50 µg/ml ascorbic acid, 100 nM dexamethasone, and 10 ng/ml transforming growth factor β). The medium was changed three times a week. After 21 days, the cells were harvested, embedded in paraffin, and stained with 1% Alcian Blue (Sigma–Aldrich, St. Louis, MO) in 5% acetic acid. The blue color in the image represents sulfated proteoglycan deposits that are indicative of functional chondrocytes.

### Nimblegen human expression assay HG18

mRNA extraction for O-ASC1, O-ASC4, O-ASC5, subcutaneous ASCs (SQ-ASCs), and BM-MSCs was performed using the Qiagen RNeasy Mini Kit (Qiagen, Valencia, CA) according to the manufacturer’s protocol. Nimblegen (Madison, WI) HG18 72 k microarrays containing 72,000 long oligonucleotides (60-mer) that tile the human genome were used for gene expression array profiling. Gene expression profiling was performed a minimum of two times for each cell lines. Microarray images were processed in NimbleScan v2.3 (Nimblegen, Madison, WI). Normalization was performed using the robust multi-array analysis algorithm with default settings.

### Expression analysis software

To perform cluster analysis of sample and expression profiling analysis of genes, the BRB Array Tools Version 4.2.1 (Richard Simon and Amy Peng Lam, National Cancer Institute, Bethesda, MD) was used. The samples were clustered using unsupervised hierarchical clustering with default settings. Class comparisons using a univariate *t*-test with a random variance model on RMA (Robust Multi-array Average) normalized data were used to identify genes that were significantly differentially expressed. A significance level of p=0.001 was designated for each univariate test. 

### Metabolic assays

Cells were plated at 50,000 cells per well in 12-well plates for 48 hours. Supernatants were collected for extracellular metabolic analysis and cell pellets were lysed with RIPA buffer for protein analysis. Glucose consumption was assessed by Wako Glucose kit and the assays were done according to manufacturer’s protocol. Cell supernatants were added to a 96-well plate filled with reconstituted Wako Glucose reagent (Wako Chemicals, Richmond, VA). Next, the plate was incubated at 37°C and was shaken simultaneously. The absorbance was detected at 505nm and 600nm by spectrophotometer (SpectraMax^®^ M5; Molecular Devices) to measure glucose contents of the samples. The results were expressed in μmol/mg of protein [9]. 

The lactate contents of the samples were measured by Trinity Lactate Kit according to manufacturer’s protocol. Briefly, cells supernatant were diluted 1:10 in PBS and then diluted samples were added to 96-well plate filled with reconstituted Trinity Lactate reagent. The prepared 96-well plate was protected from light and incubated at 37°C for one hour. The absorbance was detected at 540nm by spectrophotometer (SpectraMax^®^ M5; Molecular Devices) to measure lactate contents of the samples. Pyruvate assay involved colorimetric nicotinamide adenine dinucleotide (NADH) quantification. Lactate dehydrogenase (LDH) catalyzes conversion of pyruvate into lactate with the expense of NADH. Briefly, NADH solution was prepared by dissolving NADH (Sigma Aldrich) in Tris solution (pH=7.0). Lactate dehydrogenase (LDH) was reconstituted in 50% glycerol and diluted in Tris solution (pH=7.0). NADH solution was added to 96-well plate prior to addition of cell supernatant. The prepared plate was incubated at 37 °C for five minutes and was shaken. The absorbance was read at 340nm by spectrophotometer (SpectraMax^®^ M5; Molecular Devices) to scan the basal levels of NADH. Next, LDH was added and the plate was protected from light and was incubated at 37 °C for one hour. The final levels of NADH were measured at the same wavelength. Loss of NADH absorbance corresponds to pyruvate contents of samples. Intracellular ATP contents were measured for the different cell types using the Cell Titer-Glo Luminescent cell viability assay (Promega). The cells were seeded in 96-well plates (white) at 8,000 cells per well for 24 hours. Twenty-four hours later, cells were incubated for 2 hours in either presence of oligomycin (2.5μg/ml) or 2-deoxyglucose (100mM). The ATP content was detected according to the manufacturer's instructions, with a spectrophotometer (SpectraMax^®^ M5; Molecular Devices).

### Proliferation assay

OVCA 429, OVCA 433 cells, A2780, and SKOV3 cells were stably transfected with firefly luciferase gene using a lentiviral method and plated at 5000 cells/well in a BD Falcon 96-well plate, alone or in 1:1 co-culture with O-ASC1 or O-ASC4. Co-cultures were performed in DMEM (Mediatech, Manassas, VA) with 10% FBS and 1% penicillin and streptomycin. After 24 hours, 0.15 mg/ml D-luciferin was added and incubated for 1 hour. Luminescence was measured with a luminometer (FLUOstar Omega, BMG Labtech, Offenburg, Germany). The media were replaced, and measurements were repeated for up to 11 days of culture.

### Migration assay

Cell migration was assayed using conditioned media (CM) generated from O-ASC1 and O-ASC4. O-ASCs were cultured on a 6-well plate to confluence in α-MEM medium (Mediatech, Manassas, VA) with 20% FBS (HyClone) and 1% penicillin, streptomycin and L-glutamine (Mediatech, Manassas, VA). After washing with PBS, serum-free DMEM, with 1% penicillin and streptomycin (Mediatech, Manassas, VA) was added to each well. After 36 hours, O-ASC CM was collected. Ovarian cancer cell lines of interest were plated (in serum-free media) in the upper portion of a 24-well transwell plate with 8 µm pores (Corning, Inc., Corning, NY) with 500 µl of O-ASC CM added to the bottom of each well. All samples were assayed in duplicate or triplicate. After 24 hours, the migrated cells were stained using Hema 3 Stat Pack (Thermo Fisher Scientific, Waltham, MA). Optical density was read directly from the 96-well plate at 590 nm from an enzyme-linked immunosorbent assay (ELISA) plate reader (BioTek Instruments, Winooski, VT).

### Radiosensitivity assay

Luciferase-labeled A2780 cells were plated (at a density of 10,000 cells/well) in a BD Falcon 96-well plate, alone or in 1:1 or 1:19 co-culture with O-ASC1 or O-ASC4. Co-cultures were performed in DMEM (Mediatech, Manassas, VA) with 10% FBS, 250 µg/ml G418, 1% penicillin and streptomycin. After 24 hours, cells were irradiated at 0 Gy, 2 Gy, 4 Gy, 6 Gy, and 8 Gy via Cesium 137 external beam radiation. Luciferase expression was measured in 0.15 mg/ml D-luciferin. After 1 hour of incubation at 37°C, luminescence was measured with a spectrophotometer (FLUOstar Omega, BMG Labtech, Offenburg, Germany). Media was then replaced with new media containing G418, and measurements were repeated every other day until cells reached confluence.

### Paclitaxel and carboplatin assays

Luciferase-labeled OVCA 429, OVCA 433, A2780, and SKOV3 cells were plated (at a density of 10,000 cells/well) in a BD Falcon 96-well plate, alone or in 1:1 co-culture with O-ASC1 and O-ASC4. Co-cultures were performed in DMEM (Meditech, Manassas, VA). After 24 hours, OVCA 429 to 0.5 μM paclitaxel 200 μM carboplatin, OVCA 433 to 1 μM paclitaxel and 100 μM carboplatin, A2780 to 20 μM paclitaxel 200 μM carboplatin and SKOV3 to 50 μM paclitaxel and 100 μM carboplatin were exposed. Luciferase expression was measured in 0.15 mg/ml D-luciferin. After 1 hour of incubation at 37°C, luminescence was measured with a luminometer (FLUOstar Omega, BMG Labtech, Offenburg, Germany). The media were then replaced with new media containing the designated concentration of paclitaxel and carboplatin and measurement were repeated over 9 days.

### O-ASCs *in-vivo* engraftment

To determine if O-ASCs engraft in ovarian cancer stroma, 5 nude mice were injected intraperitoneally (IP) with 5 x 10^6^ luciferase expressing SKOV-3 tumor cells with or without the same number of RFP-labeled O-ASC1. The control group consisted of mouse injected with only O-ASC1. Tumor growth was monitored with *in-vivo* bioluminescent imaging. Mice were sacrificed after 53 days. All mice were euthanized by protocol approved by MD Anderson’s Institutional Animal Care and Use Committee (IACUC). Tumor and ovaries were fixed and paraffin embedded for histologic analysis. Immunofluorescence was performed on ovaries in order to detect RFP expressing O-ASC in all mice. All animal work is conducted according the approved mouse protocol at MD Anderson Cancer Center. Mouse protocol was approved by MD Anderson's Institutional Animal Care and Use Committee (IACUC). Hematoxylin and eosin (H&E) staining was performed using standard protocol on slides of paraffin embedded tissues. 

### Statistical analysis

T-tests were performed using GraphPad Prism software, version 5.00 (GraphPad Software, San Diego, CA, www.graphpad.com) to determine statistically significant differences between groups. Differences were considered significant if *p* < 0.05.. All graphs show mean ± SEM.

## Results

### Characterization of O-ASCs

O-ASCs were isolated from three patients with ovarian cancer. O-ASC1 was obtained from omental adipose tissue of a patient with endometrial and ovarian adenocarcinoma without omental metastasis. O-ASC4 and O-ASC5 were isolated from grossly normal appearing omentum obtained from a patient with serous ovarian carcinoma with omental metastasis. Differentiation assays demonstrated that O-ASCs, like BM-MSCs, differentiate into adipogenic, chondrogenic, and osteogenic mesenchymal lineages (Figure S1). Cell surface markers were characterized with flow cytometry (Figure S2). O-ASC1 and O-ASC4, similar to BM-MSCs, expressed CD29, CD44, CD73, CD90, and CD105. They did not express CD11b, CD34, CD45, or EpCam. The surface marker CD34 (transmembrane glycoprotein expressed in early hematopoietic and vascular tissue) was expressed by 10% of O-ASC1s compared with 0.2% of BM-MSCs and 1.42% of O-ASC4. Endoglin (CD105) and CD44 were nearly universally expressed in BM-MSCs (99.34% and 100% of cells, respectively) but were expressed at lower levels on O-ASC1 (87.24% and 46.1% of cells, respectively), or O-ASC4 (7.98% and 30% of cells, respectively). Other markers were expressed at similar levels in all cell lines. 

### Gene expression

**Figure 1:**
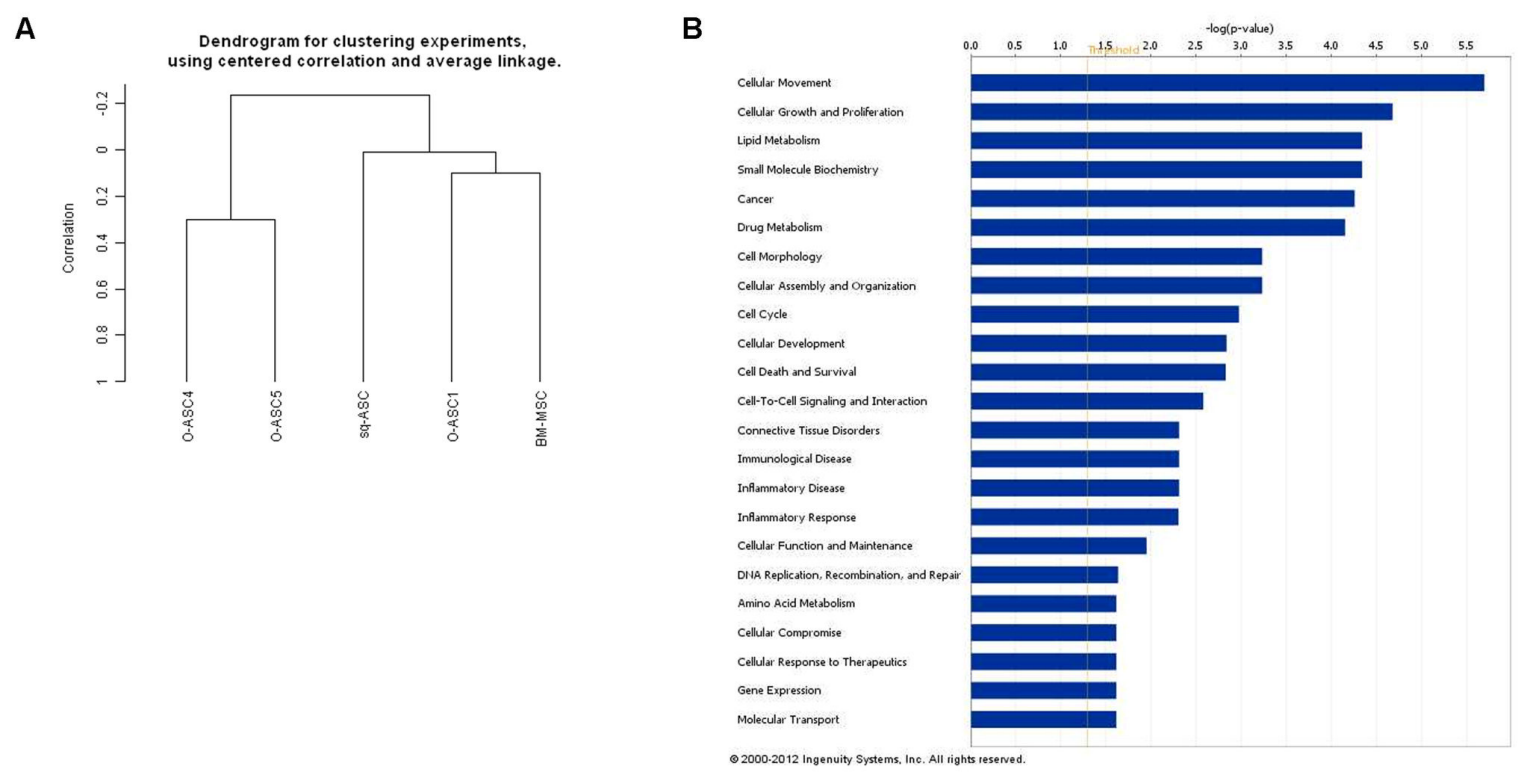
Gene expression profiling of O-ASC as compared to BM-MSC and SC-ASC. Unsupervised clustering was performed to generate a dendrogram using center correlation and average linkage (A) (O-ASC1, O-ASC4, O-ASC5, BM-MSC: n = 2; SC-ASC: n = 4). IPA analysis revealed pathways with significantly different expression between O-ASC1 and O-ASC4 (B). Functions are listed from most significant (higher bars) to least significant (lower bars), and the perpendicular yellow line denotes the threshold for significance (P=0.05).

Gene expression of O-ASC1, O-ASC4, O-ASC5 for comparison with SQ-ASC and BM-MSC was surveyed with Nimblegen arrays. A hierarchical clustering algorithm was used to group samples on based on similarity in the expression of all 24,000 genes. Dendrograms based on unsupervised clustering separated samples into two main branches; 1) O-ASC4 and O-ASC5, which were isolated from patients with metastasis into the omentum, and 2) SQ-ASCs and O-ASC1) and BM-MSCs (Figure 1A). 

Using a package of statistical tools, we identified 415 significantly differentially expressed genes between O-ASC1 and O-ASC4. Fifty-six percent of over-expressed genes (minimum 10x-fold change) in O-ASC4 relative to O-ASC1 coded for products whose final localization is in the plasma membrane or extracellular space. Ingenuity Pathways Analysis (IPA) analysis demonstrated that most of these genes are involved in cellular movement, growth, and proliferation and cancer or small molecule metabolism (Figure 1B). Table 1 lists the top 25 genes (fold change cut-off of >4 and p value < 0.001) whose expression significantly differed between OSC4 and OSC1. 

**Table 1:** List of 25 genes whose expression was statistically significantly different between O-ASC4 vs. O-ASC1.

**Symbol**	**Accession number**	**Entrez gene name**	**Location**	**Type**	**Fold change**	**Parametric p-value**
ACAN	NM_001135, NM_013227	Aggrecan	Extracellular space	Other	116.51	1.20E-06
VNN1	NM_004666	Vanin 1	Plasma membrane	Enzyme	0.032	7.20E-06
GPR116	NM_015234	G protein-coupled receptor 116	Plasma membrane	G-protein coupled receptor	23.81	1.02E-05
GRIK2	NM_021956, NM_175768	Glutamate receptor, ionotropic, kainate 2	Plasma membrane	Ion channel	21.19	1.02E-05
NRCAM	NM_001037132	Neuronal cell adhesion molecule	Plasma membrane	Other	0.033	1.13E-05
C1QL3	NM_001010908	Complement component 1, q subcomponent-like 3	Extracellular space	Other	0.042	1.24E-05
ITGA8	NM_003638	Integrin, alpha 8	Plasma membrane	Other	51.01	1.35E-05
CADM3	NM_021189	Cell adhesion molecule 3	Plasma membrane	Other	0.049	1.51E-05
NEBL	NM_006393	Nebulette	Plasma membrane	Other	0.058	2.17E-05
GJA5	NM_005266	Gap junction protein, alpha 5, 40 kDa	Plasma membrane	Transporter	24.45	2.27E-05
NPTX1	NM_002522	Neuronal pentraxin I	Extracellular space	Other	35.6	2.51E-05
AIF1L	NM_001002260	Allograft inflammatory factor 1-like	Plasma membrane	Other	12.84	2.73E-05
F11R	NM_016946	F11 receptor	Plasma membrane	Other	0.098	3.18E-05
PF4	NM_002619	Platelet factor 4	Extracellular space	Cytokine	0.097	3.39E-05
OXTR	NM_000916	Oxytocin receptor	Plasma membrane	G-protein coupled receptor	14.55	3.63E-05
PCDH10	NM_032961	Protocadherin 10	Plasma membrane	Other	57.29	3.83E-05
SGCA	NM_000023	Sarcoglycan, alpha (50 kDa dystrophin-associated glycoprotein)	Plasma membrane	Other	10.43	4.00E-05
SORBS1	NM_001034954	Sorbin and SH3 domain containing 1	Plasma membrane	Other	30.08	4.00E-05
CXCL5	NM_002994	Chemokine (C-X-C motif) ligand 5	Extracellular space	Cytokine	0.12	4.15E-05
ESM1	NM_007036	Endothelial cell-specific molecule 1	Extracellular space	Growth factor	20.67	4.34E-05
CCBP2	NM_001296	Chemokine binding protein 2	Plasma membrane	G-protein coupled receptor	0.13	4.39E-05
IL13RA2	NM_000640	interleukin 13 receptor, alpha 2	Plasma membrane	Transmembrane receptor	0.068	4.60E-05
MMP7	NM_002423	Matrix metallopeptidase 7 (matrilysin, uterine)	Extracellular space	Peptidase	0.1	4.68E-05
WFDC1	NM_021197	WAP four-disulfide core domain 1	Extracellular space	Other	19.45	4.75E-05

To calculate the statistical significance, we used Student's *t*-test.

### Metabolic assays

**Figure 2:**
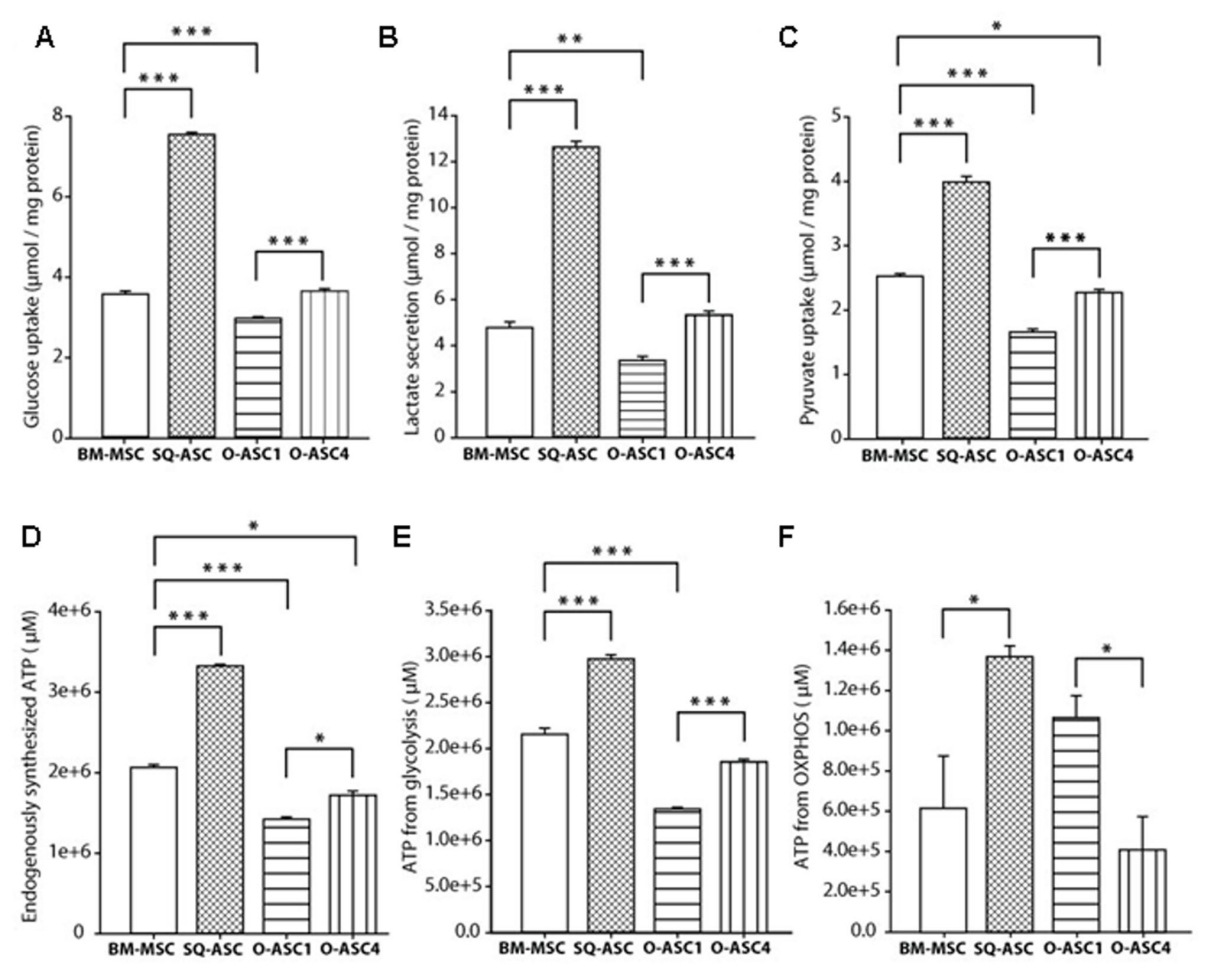
Metabolic characterization of bone marrow-derived and adipose derived mesenchymal stem cells. Metabolic analysis was performed on BM-MSC, SC-ASC and O-ASC1 and 4, including glucose uptake (A), lactate secretion (B), pyruvate uptake (C), endogenous ATP production (D), ATP derived from glycolysis (E) and ATP derived from oxidative phosphorylation (OXPHOS)(F). Data are presented as mean ± S.E.M. *p<0.05, **p<0.01 and ***p< 0.001 for unpaired 2-tailed Student’s t-test (n = 8).

Stroma has been shown to promote malignant progression by altering cancer cell metabolism. According to the “reverse warburg effect”, stromal cells upregulate glycolysis, increasing lactate production that is secreted and consumed by adjacent cancer cells to fuel oxidative phosphorylation (OXPHOS) [10].  We hypothesized that the pro-proliferative and migratory effects of OSC may be accounted for by metabolic differences in stromal isolates. SQ-ASC had the highest level of glucose uptake and lactate secretion compared to BM-MSC and O-ASCs (Figure 2A and 2B).  Among O-ASCs isolates, O-ASC4 had significantly higher rate of glycolysis and lactate secretion than O-ASC1 (Figure 2A and 2B). O-ASC4 had higher rate of pyruvate uptake than O-ASC1 which is likely converted to lactate given that the have a higher rate of glycolytic activity in O-ASC4 (Figure 2C).  Steady state ATP production was higher in O-ASC4 than O-ASC1 (Figure 2E-F). The ATP production attributed to glycolysis was determined by inhibiting the F1F0-ATPsynthase with oligomycin while ATP from oxidative phosphorylation (OXPHOS) was measured by inhibiting the glycolysis pathway with 2-deoxyglucose (2DG).   These results demonstrated that O-ASC4 produced ATP preferentially from glycolysis while O-ASC1 produced ATP from OXPHOS (Figure 2 E and F).

### O-ASCs effects on ovarian cancer cell proliferation

**Figure 3:**
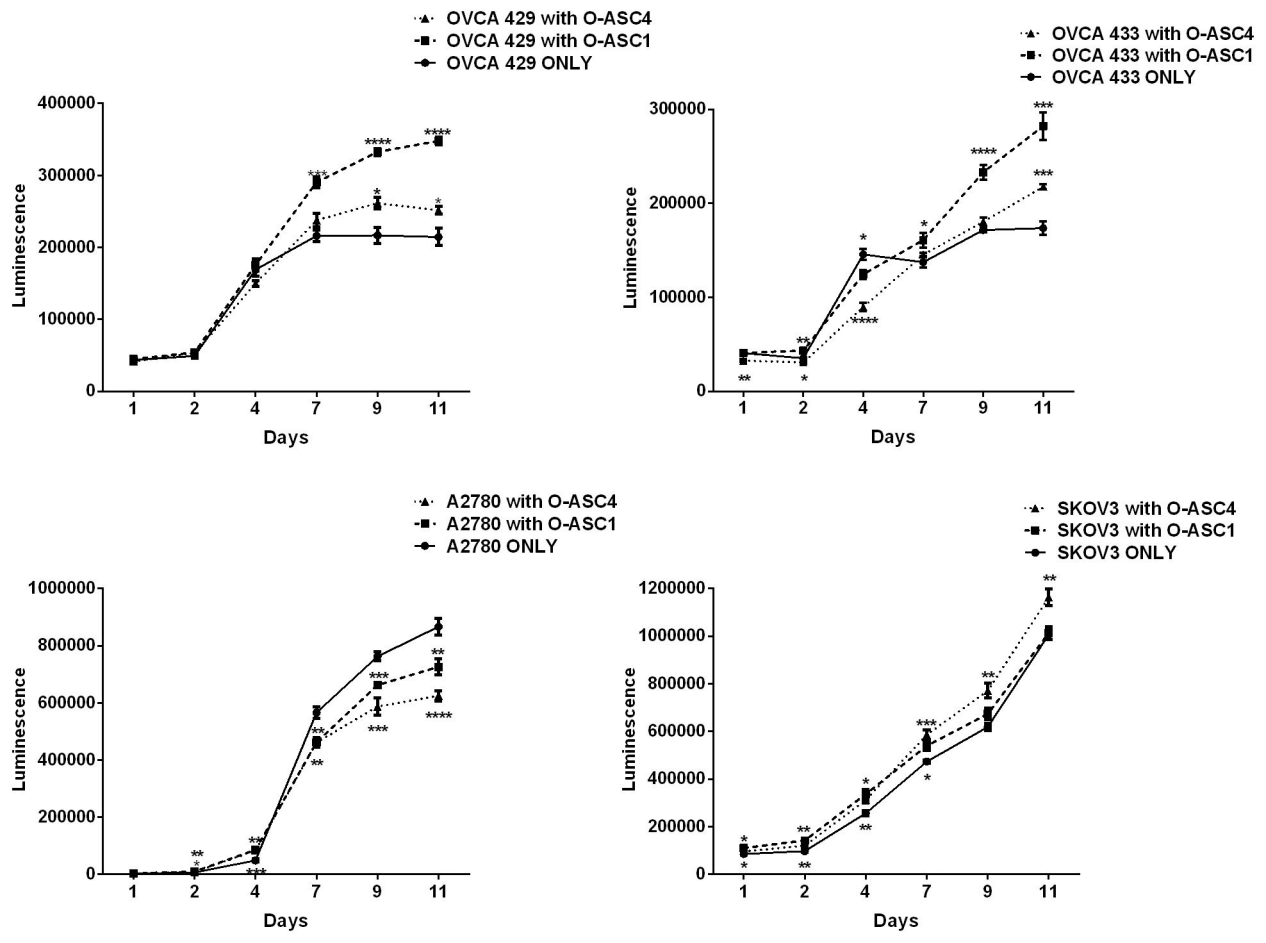
*In-vitro* effects of O-ASC on the proliferation of multiple ovarian cancer cell lines. Luciferase expressing ovarian cancer cell lines were cultured alone or in a 1:1 ratio with unlabeled O-ASCs. Significant difference between controls (cancer cells cultured alone) and cancer cells co-cultured with O-ASCs are shown: *, P < 0.05; **, P < 0.01; ***, P < 0.001; ****, P < 0.0001 (n = 5). ANOVA with a Bonfferoni multiple comparisons test.

Given the distinction between O-ASC1 and O-ASC4 with regard to gene expression analysis and metabolic assays, we hypothesized that these isolates may have distinct effects on ovarian cancer biology. To test this, the effects of O-ASC1 and O-ASC4 on ovarian cancer cell proliferation, migration and chemsensitivity were compared. First, to determine if O-ASCs influence ovarian cancer cell metabolism, unlabeled O-ASCs and luciferase-expressing ovarian cancer cell lines (OVCA 429, OVCA 433, A2780, and SKOV3) were co-cultured at a 1:1 ratio until cell became confluent 11 days after plating. OVCA 429 and OVCA 433 cells proliferation was significantly increased at 7 (p<0.001 and p<0.5 respectively), 9 (p<0.0001 for both cell lines) and 11 days (p<0.0001 and p<0.001 respectively) when co-cultured with O-ASC1 as compared to control. Significant pro-proliferative effects of O-ASC4 were seen in OVCA 429 cells after 9 days (p<0.01) and OVCA 433 cells at 11 day of co-culture (p<0.001). O-ASC4 increased proliferation of SKOV3 cells at 7 (p<0.001), 9 (p<0.01) and 11 day (p<0.01). Interestingly, O-ASC1 and O-ASC4 suppressed the proliferation of A2780 human ovarian carcinoma cells (p<0.01 on days 7, 9, and 11) (Figure 3). To evaluate the impact of a lower ratio of ASC cells, we performed proliferation assays with a 1:19 O-ASCs:cancer cells ratio (Figure S3). There was no significant difference in proliferation rate for OVCA 429, OVCA 433, A2780 and SKOV3 cell lines when co-cultured with O-ASCs at this ratio. 

### O-ASCs effects on ovarian cancer cell migration

**Figure 4:**
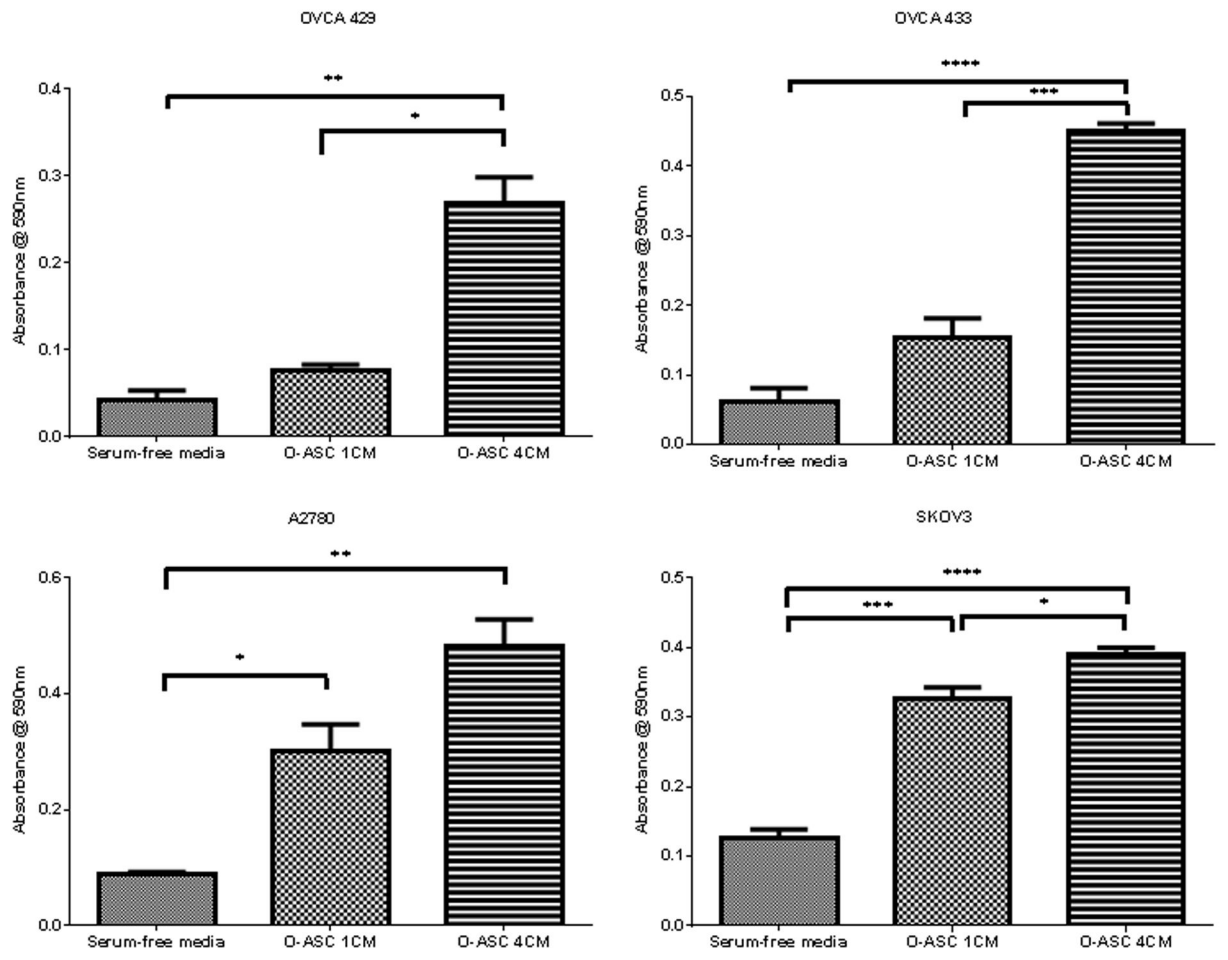
O-ASC increase the migration of ovarian cancer cells. Ovarian cancer cells migrated through a 8 μm pore in the presence of control of O-ASC conditioned media. The absorbance at 590 nm reflects the number of cells that passed through the transwell membrane. Significant differences are shown as follows: *, P < 0.05; **, P < 0.01; ***, P < 0.001 for unpaired 2-tailed Student’s t-test (n = 3).

To investigate the impact of O-ASCs on ovarian cancer cell migration, modified boyden chamber assays were performed with conditioned media (CM) from O-ASCs. The number of migrated cancer cells (OVCA 429, OVCA 433, A2780, and SKOV3) significantly increased following incubation with CM from both O-ASC1 and 4. O-ASC4 CM significantly increased migration of OVCA 429, 433 and A2780 cells as compared to O-ASC1. (Figure 4, Figure S4).

### O-ASCs effect on ovarian cancer cell response to chemotherapy and radiation treatment

**Figure 5:**
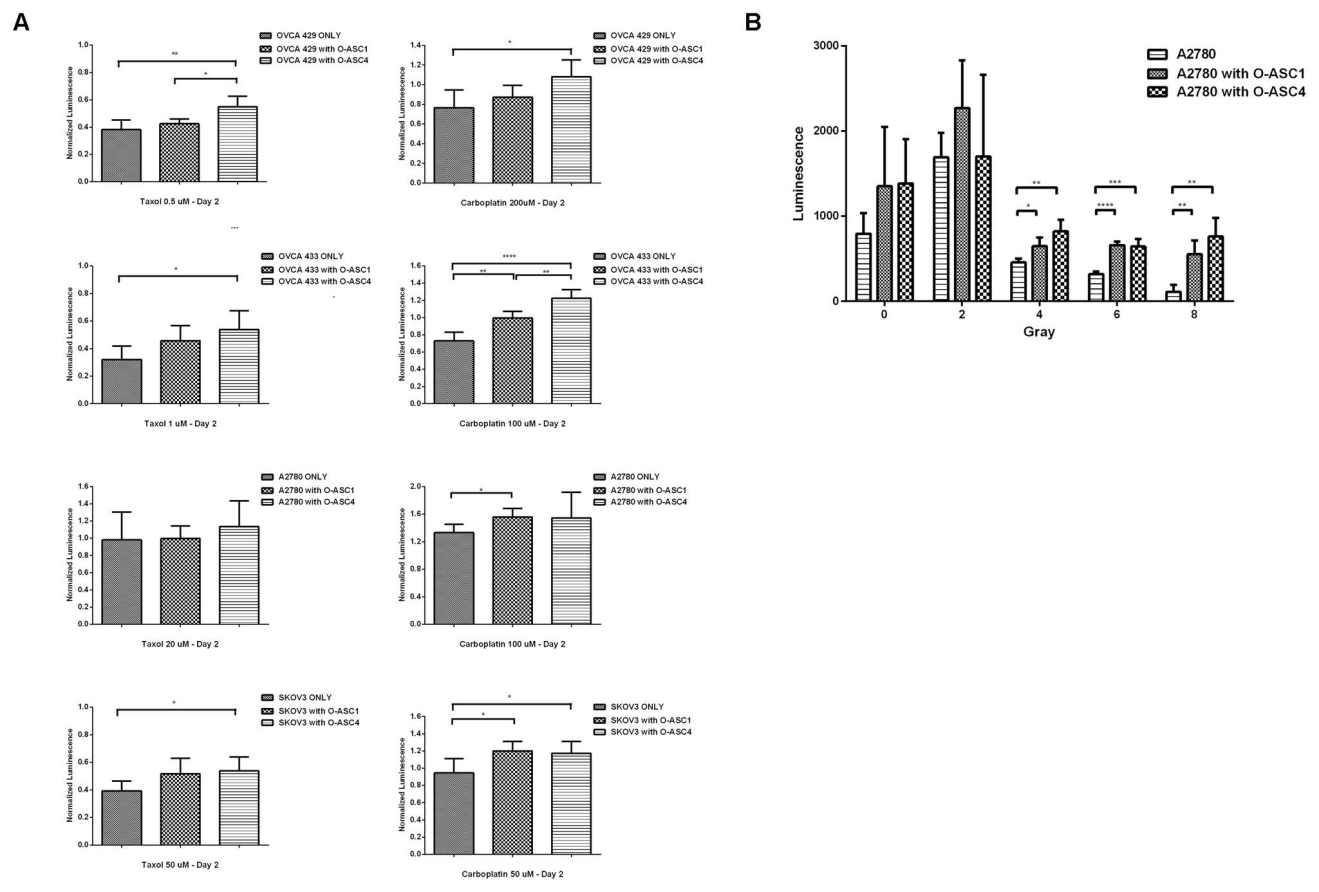
Chemoprotective and radioprotective effect of O-ASCs on ovarian cancer cell lines. Ovarian cancer cells were cultured with or without an equal number of O-ASC and treated with the doses of paclitaxel and carboplatin (n = 5) (A) or radiation (n = 4) 0-8 Gray (B) as shown. Significant differences are shown as follows: *, P < 0.05; **, P < 0.01; ***, P < 0.001; ****, P < 0.0001 for unpaired 2-tailed Student’s t-test.

To determine whether the presence of O-ASCs impact ovarian cancer cells response to chemotherapy, we treated cells alone and co-cultured with O-ASCs with paclitaxel or carboplatin. OVCA 429 cells had significantly greater survival after treatment with carboplatin or taxol when co-cultured with O-ASC4. OVCA 433 and SKOV-3 survival after treatment of carboplatin and taxol increased with co-culture of both O-ASC1 and O-ASC4. A2780 survival was not impacted by O-ASC co-culture (Figure 5A). To determine O-ASCs effect on radiation sensitivity, A2780 cells were co-cultured 1:1 for 7 days with O-ASC1 or O-ASC4. After incubation, cells were irradiated at 2, 4, 6, and 8 Gy. We observed a significant increase in survival after 4, 6, and 8 Gy in A2780 cells co-cultured with O-ASC1 (p<0.05, p<0.0001 and p<0.01, respectively) and O-ASC4 (p<0.01, p<0.001, and p<0.01, respectively) compared with the control (Figure 5B). The radio-protective role of adipose stem cells was not clearly shown in a similar experiment performed with 1:19 ratio of O-ASCs cells to. cancer cells (Figure S5).

### O-ASC engraftment

**Figure 6:**
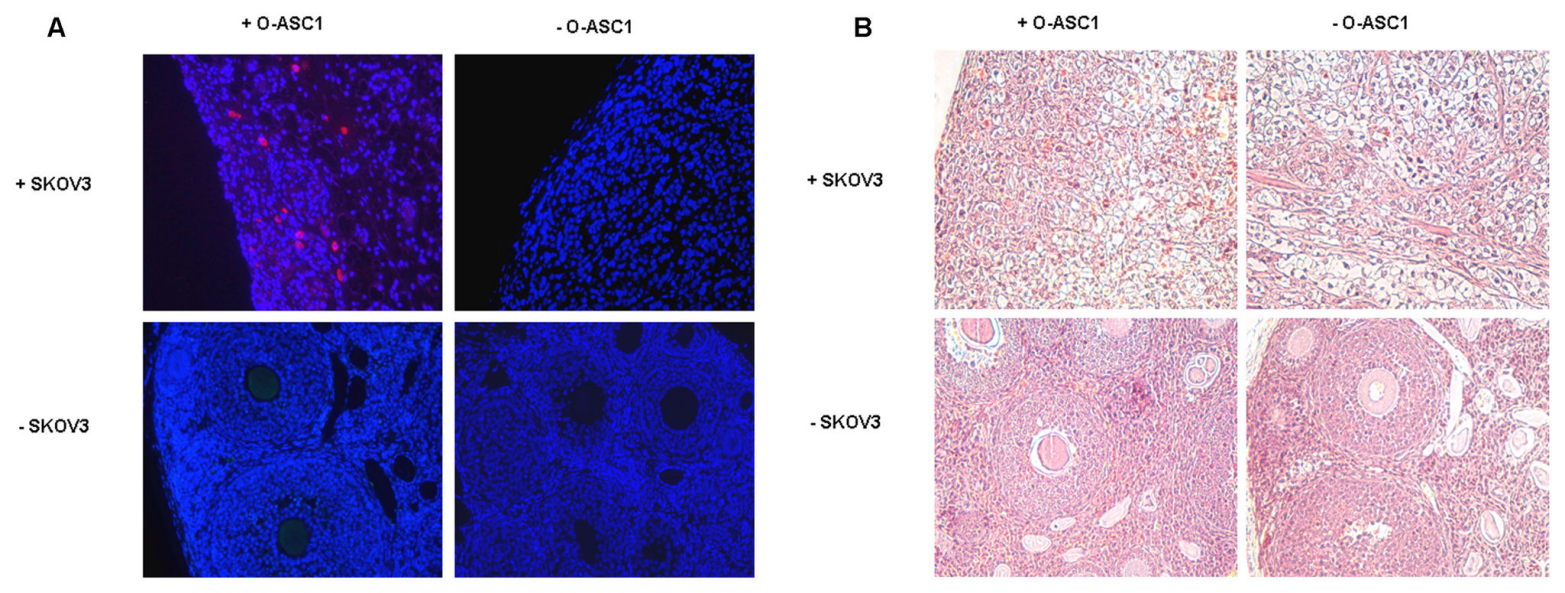
O-ASC engraft in ovarian xenografts but not normal ovary. (A) Immunoflouresence was performed to detect O-ASC in ovarian SKOV3 xenografts and grossly normal ovary. O-ASC are detected as red cells within the stroma of ovarian tumors in mice injected with RFP expressing O-ASC. (B) H&E tissue staining (20x magnification).

Next, we aimed to determine if O-ASCs engraft in ovarian cancer stroma and normal tissues, including ovary. Nude mice were injected intraperitoneally with luciferase expressing SKOV-3 tumor cells with or without an equal number of RFP-labeled O-ASC1. Tumor growth was monitored with *in-vivo* bioluminescent imaging. After 7 days, luciferase activity was significantly higher in mice treated with O-ASCs but subsequently declined (Figure S6). O-ASCs were re-injected on day 25. Immunofluorescence was performed 7 days later to detect RFP expressing O-ASCs within ovarian cancers and uninvolved ovary. RFP expressing O-ASCs were detected within the stroma of ovarian xenografts but not within uninvolved ovaries (Figure 6A, 6B), demonstrating tumor-specific engraftment, as has been reported with MSC from other sources.

## Discussion

In addition to adipocytes, the omentum contains blood and lymphatic vessels as well as a rich inflammatory infiltrate which make it distinct from subcutaneous adipose tissue. We have shown that O-ASCs from ovarian cancer patients share features such as differentiation potential and cell surface marker expression with MSCs and ASCs from other sites ([8] and Figure S1 and 2). Analysis of surface marker comparison of these specific tissue resident MSCs populations revealed small differences in the expression of CD34, CD44, and CD105 markers [11-14] (Figure S2). These differences in lineage markers may reflect the different differentiation potential of ASCs and MSCs which has been shown to occur within tumors and normal tissues [15]. Clustered gene expression data from microarrays confirmed that ASCs are similar to BM-MSCs (Figure 1A). Interestingly, O-ASCs from patients with ovarian cancer metastasis clustered together while O-ASC from a patient without metastasis was more similar to BM-MSCs and SQ-ASCs. Regulation of ovarian cancer cell lines by MSC populations varied across isolates with some evidence for greater effects on tumor cells from O-ASCs derived in the setting of omental metastases. We found that O-ASCs, like BM-MSCs, engraft in tumor stroma, but not normal ovary [8].

O-ASC4 promoted tumor cell migration and chemotherapy resistance more than O-ASC1, suggesting that ASCs have heterogenous effects on ovarian cancer. These distinct phenotypes may be attributed to characteristics of the individual, such as obesity or due to exposure to tumor derived growth factors, from whom they are isolated [16,17] or other intrinsic tumor promoting characteristics in the stroma that contributed to the development of omental metastses. This suggests that O-ASCs phenotype may thus be an unappreciated factor influencing clinical outcome. 

O-ASCs may contribute to the high rate of ovarian cancer metastasis to the omentum [18]. BM-MSCs have been shown to promote cancer cells ability to grow, survive, and migrate by secreting a broad variety of cytokines, chemokines, and growth factors [3-6]. In migration experiments, conditioned media from O-ASCs promoted cancer cell migration, supporting by paracrine mechanism of ASCs (Figure 4 and Figure S3). O-ASC4 conditioned promoted cancer cell migration significantly more than O-ASC1 conditioned media for three of the four ovarian cancer cell lines examined. Comparative analyses of gene expression profiles between O-ASC4 and O-ASC1 revealed 415 differentially expressed genes. Fifty-six percent of these genes are located on the plasma membrane or are secreted into the extracellular space, which may contribute to the paracrine effects of O-ASCs. Differentially expressed pathways between O-ASC4 and O-ASC1 include: cell movement and proliferation, cell-to-cell signaling, and cytokine-mediated signaling pathways (tumor necrosis factor and interleukin-8 pathways) (Figure 1B). 

One highly overexpressed gene in O-ASC4 is ACAN gene-encoded aggrecan protein (Table 1). Aggrecan is a proteoglycan that belongs to a class of extracellular macromolecules that are necessary for the growth of multicellular structures, including tumors. Aggrecan expression is enhanced in many cancers [19] and has been reported to influence ovarian cancer growth and metastasis. One of the most highly upregulated genes in O-ASC4 is endothelial cell-specific molecule 1, also called endocan. This secreted glycoprotein is upregulated in tumor-associated lymphatic endothelial cells (LECs) [20] and has an established role in adhesion, migration, and angiogenesis [21]. Matrilysin, also known as matrix metalloproteinase-7, was expressed more highly on O-ASC4 and is known to be involved in the breakdown of the extracellular matrix in normal physiological and disease processes, such as metastasis. Matrix metalloproteinase-7 protein has been proposed as an early detection biomarker for ovarian cancer [22]. These genes and others identified are candidates for mediating the effects of O-ASC on ovarian cancer. 

Higher levels of lactate dehydrogenase and IGF-1 in O-ASC4 may contribute to the higher rate of ATP production and glycolysis observed in our metabologic assays. Lactate dehydrogenase C (LDHC) catalyzes the conversion of NAD and L-lactate to NADH and pyruvate as the starting substrate for the citric acid cycle. Two different transcript variants (1 and 2) have been detected that are over-expressed in O-ASC4 (fold change: 6.16 and 8.61; p-Value: 0.0002646 and 0.0002728 respectively). Insulin-like growth factor I (IGF-I, which has an important role in the regulation of glucose uptake, utilization and energy metabolism was also over-expressed in O-ASC4 cells (fold change: 12.71 and p-Value: 0.000307) [23,24]. 

In some cases, we observed heterogeneous effects of O-ASC on ovarian cancer cell lines. For examples, we observed that O-ASCs significantly increased the growth rate of OVCA 429, OVCA 433, and SKOV3 cells but surprisingly decreased the rate of A2780 cells. Heterogeneous effects of MSC and ASC in different tumor models have been reported previously [3]. O-ASC effects on cancer are clearly complex and dependent on signaling pathways within the stroma as well as cancer cells.

We hypothesized that O-ASC may influence ovarian cancer metabolism which could account for the effects of O-ASC on ovarian cancer proliferation and chemotherapy response. We found that O-ASC4 secreted more lactose than O-ASC1 and derived ATP preferentially from glycolysis. This is consistent with the proposed “reverse Warburg effect” in which stromal derived lactate is consumed by adjacent cancer cells to fuel oxidative phosphorylation.  Future studies will be needed to determine if enhanced lactate secretion by O-ASC4 can account for the effects of O-ASC4 on malignant cell survival.

Our experiments clearly revealed that O-ASCs represent phenotypically, functionally, and biochemically different subpopulations, depending on the cells source. They have significant effects on ovarian cancer proliferation, chemosensitivity and radio-sensitivity. Interestingly, O-ASC4 had more potent effects on proliferation, migration and chemosensitivity than O-ASC1 under many conditions. This may be due to the fact that they were isolated from a patient with a higher BMI and omental metastasis, or the altered phenotype of O-ASC4 may be due to other factors that contribute to individual heterogeneity in the ASC population. Future studies will focus on whether O-ASCs tumor-promoting effects can be attributed to clinical or disease-related characteristics.

## Supporting Information

Figure S1 **Differentiation potential of BM-MSCs and O-ASCs.** Morphology of O-ASC and BM-MSC was similar. Oil red O staining was performed to detect the formation of neutral lipid vacuoles following adipocyte differentiation. Accumulated extracellular calcium deposits were stained using Alizarin Red S after osteocyte differentiation assays. The deposition of glycosaminoglycan in chondrogenic differentiation was indicated by Alcian blue staining.
(TIF)

Figure S2 **Characterization of cell surface marker expression of BM-MSCs, O-ASC1, and O-ASC4.** Flow cytometry was performed to detect CD11b, CD29, CD34, CD44, CD45, CD73, CD90, CD105, and EpCam surface markers. The grey histogram represents the unlabeled control cells for comparison with those labeled for each surface marker (black histogram).
(TIF)

Figure S3 ***In-vitro* effects of O-ASC on the proliferation of multiple ovarian cancer cell lines.** Luciferase expressing ovarian cancer cell lines were cultured alone or in a 1:19 (O-ASCs/cancer cells) ratio with unlabeled O-ASCs (n=5).
(TIF)

Figure S4 **O-ASCs increase migration of ovarian cancer cells.** Migrated OCVA 429, OVCA 433, A2780, and SKOV3 in response to conditioned mediation from O-ASC1 or O-ASC4 (20x magnification).
(TIF)

Figure S5 **Radioprotective effect of O-ASCs on ovarian cancer cell lines.** Ovarian cancer cells were cultured with or without O-ASC in ratio 20:1 (cancer cells/O-ASCs) and treated with radiation (n = 5) 0-8 Gray.
(TIF)

Figure S6 ***Invivo* growth of SKOV3 tumors with and without O-ASC.** Nude mice were injected with 5 x 10^6^ luciferase expressing SKOV3 cells (n = 4) with and without the same number of O-ASC (n = 5). Tumor growth was monitored with bioluminescent imaging.
(TIF)
