# Supplementary figures and images for: Human Omental-Derived Adipose Stem Cells Increase Ovarian Cancer Proliferation, Migration, and Chemoresistance
Source: PLoS One. 2013 Dec 2;8(12):e81859. doi: 10.1371/journal.pone.0081859 (PMC3847080; doi:10.1371/journal.pone.0081859)

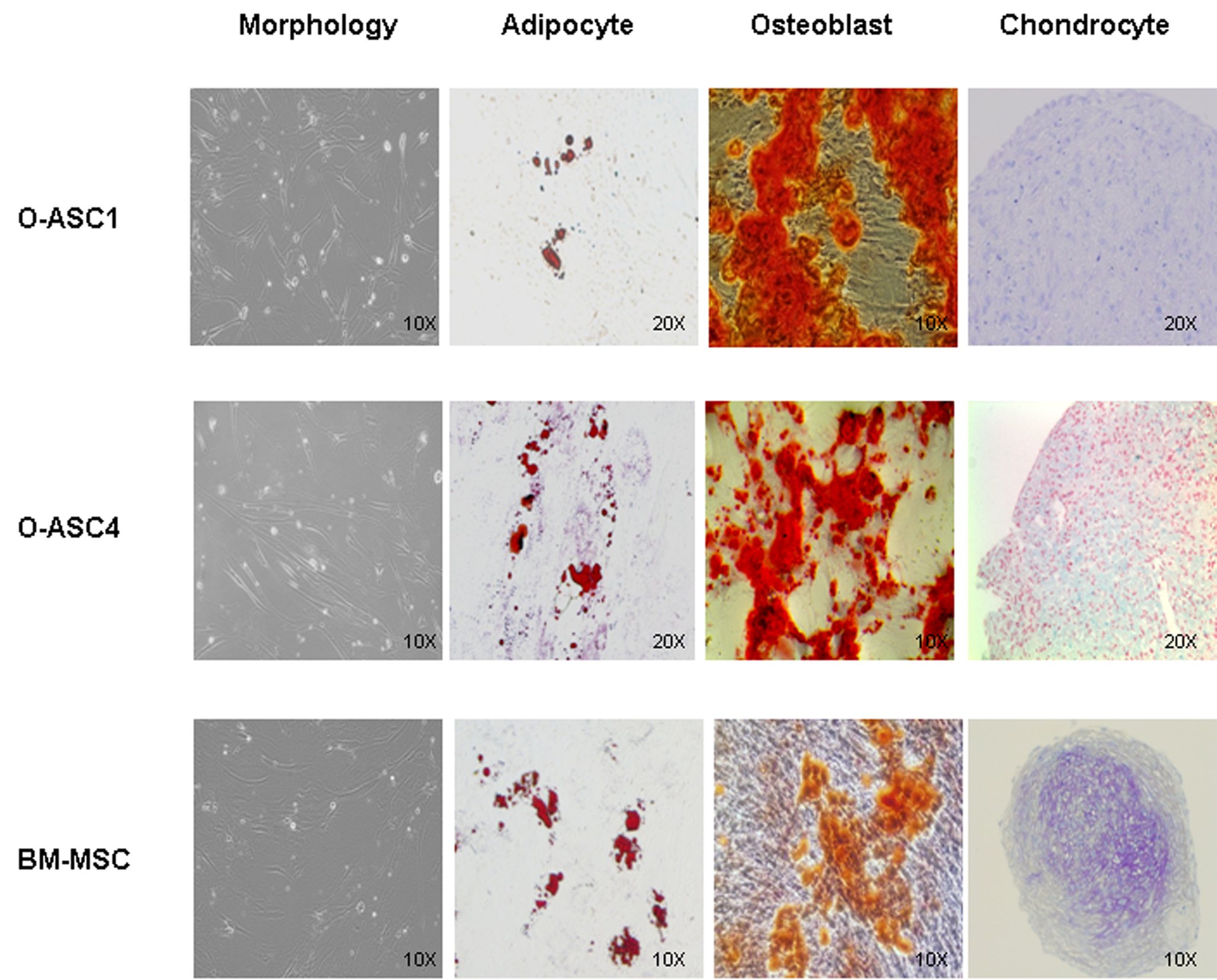

Supplement: Figure S1 — Differentiation potential of BM-MSCs and O-ASCs. Morphology of O-ASC and BM-MSC was similar. Oil red O staining was performed to detect the formation of neutral lipid vacuoles following adipocyte differentiation. Accumulated extracellular calcium deposits were stained using Alizarin Red S after osteocyte differentiation assays. The deposition of glycosaminoglycan in chondrogenic differentiation was indicated by Alcian blue staining. (TIF) [file pone.0081859.s001.tif]

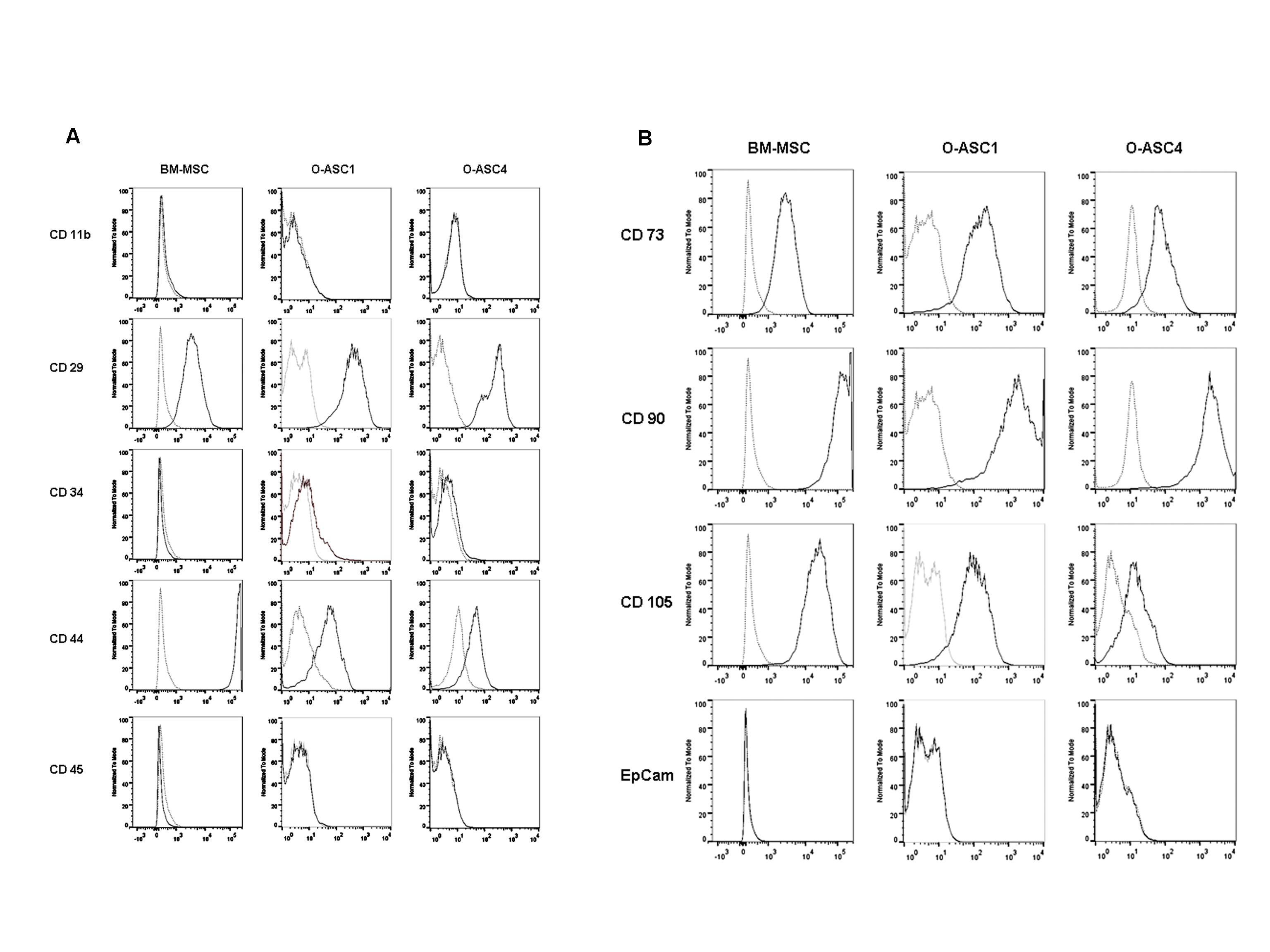

Supplement: Figure S2 — Characterization of cell surface marker expression of BM-MSCs, O-ASC1, and O-ASC4. Flow cytometry was performed to detect CD11b, CD29, CD34, CD44, CD45, CD73, CD90, CD105, and EpCam surface markers. The grey histogram represents the unlabeled control cells for comparison with those labeled for each surface marker (black histogram). (TIF) [file pone.0081859.s002.tif]

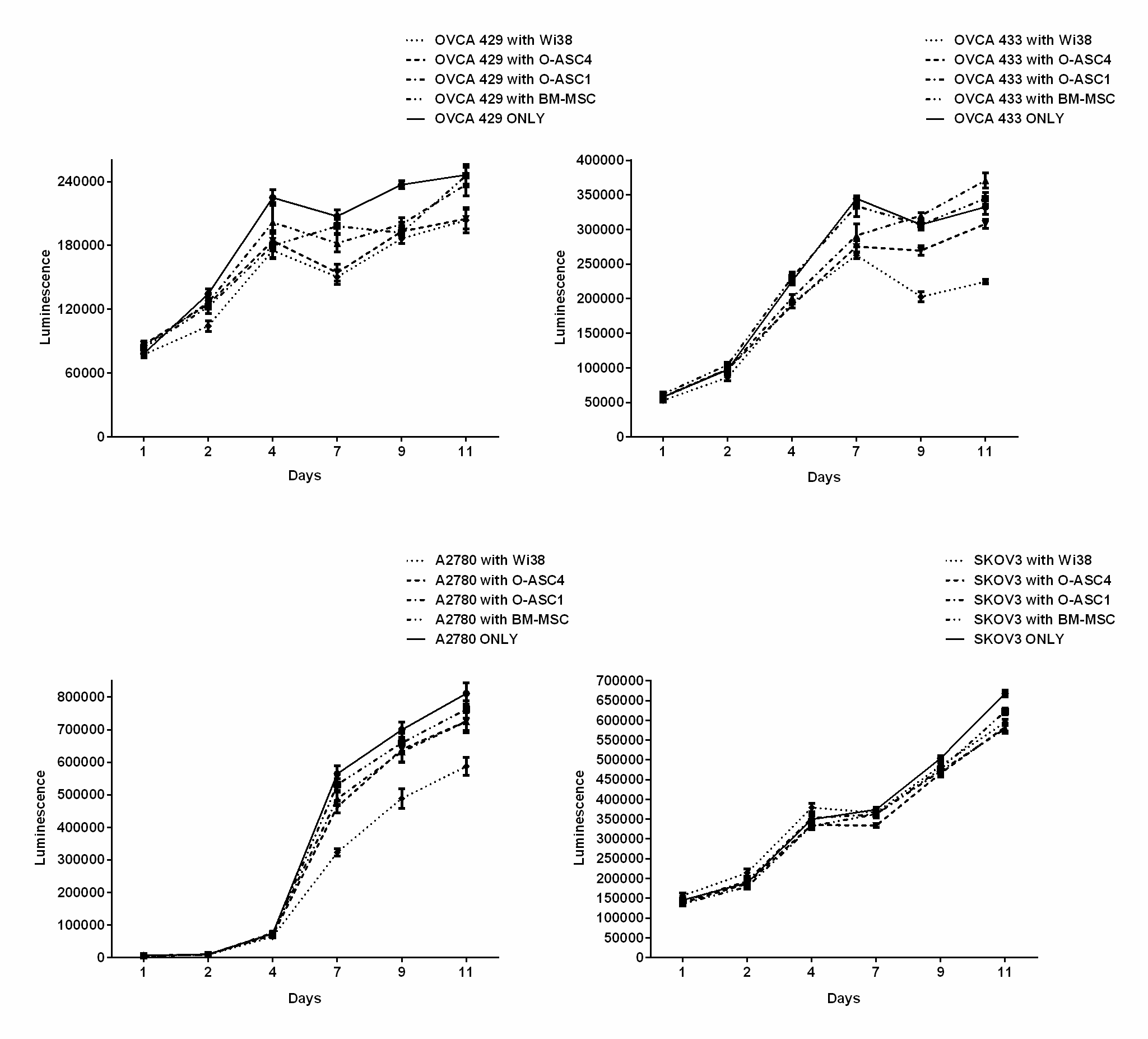

Supplement: Figure S3 — In-vitro effects of O-ASC on the proliferation of multiple ovarian cancer cell lines. Luciferase expressing ovarian cancer cell lines were cultured alone or in a 1:19 (O-ASCs/cancer cells) ratio with unlabeled O-ASCs (n=5). (TIF) [file pone.0081859.s003.tif]

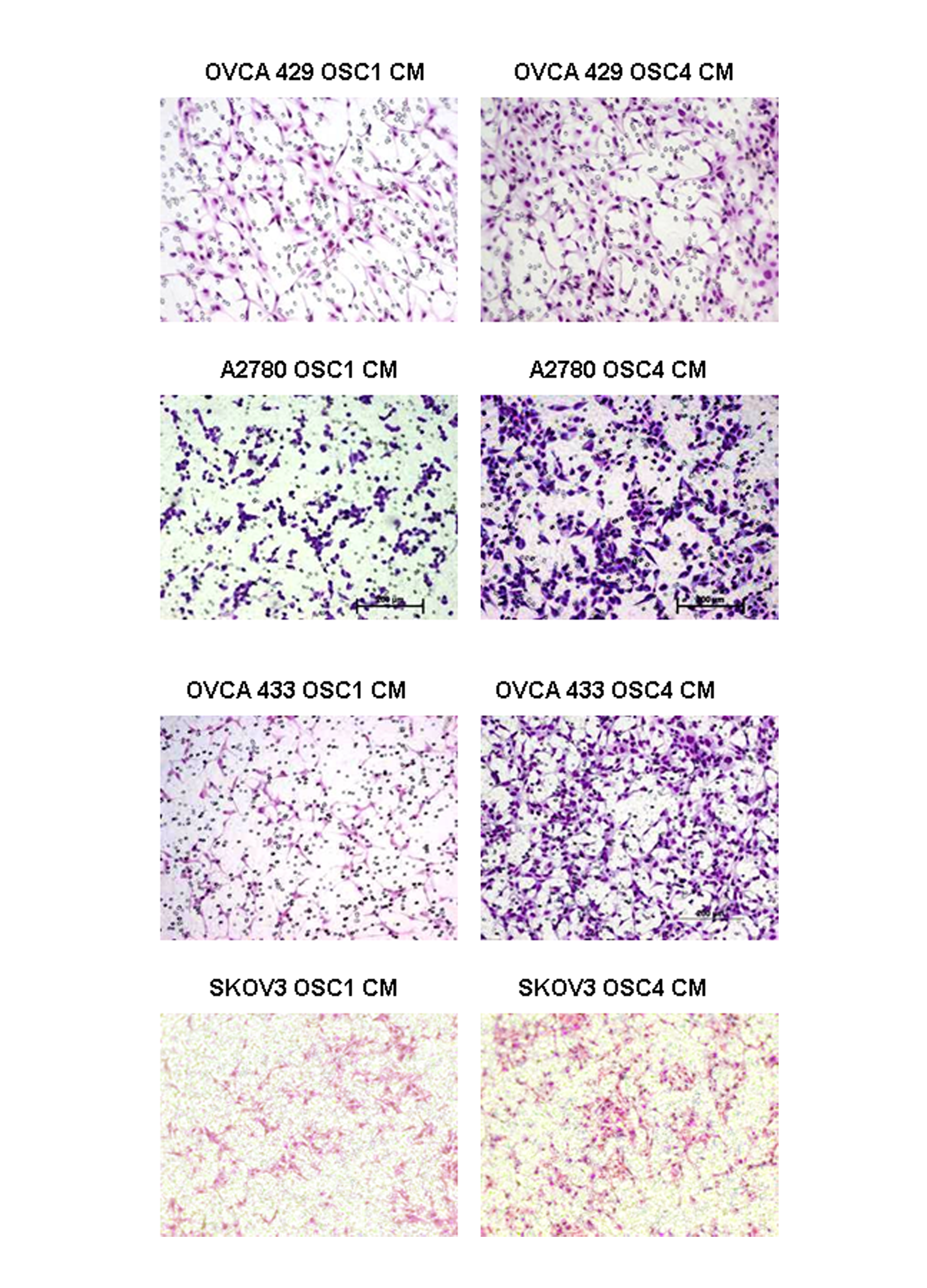

Supplement: Figure S4 — O-ASCs increase migration of ovarian cancer cells. Migrated OCVA 429, OVCA 433, A2780, and SKOV3 in response to conditioned mediation from O-ASC1 or O-ASC4 (20x magnification). (TIF) [file pone.0081859.s004.tif]

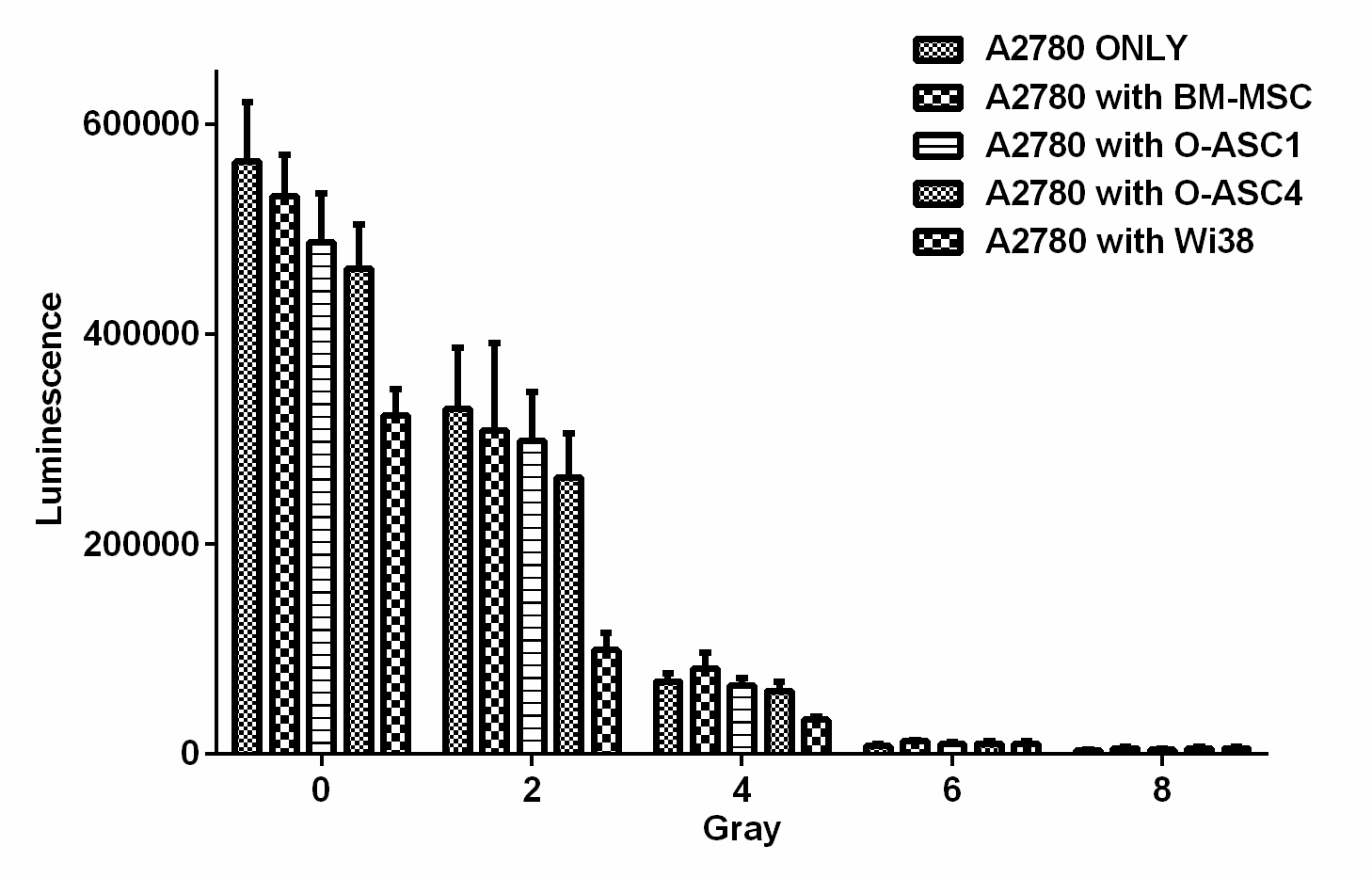

Supplement: Figure S5 — Radioprotective effect of O-ASCs on ovarian cancer cell lines. Ovarian cancer cells were cultured with or without O-ASC in ratio 20:1 (cancer cells/O-ASCs) and treated with radiation (n = 5) 0-8 Gray. (TIF) [file pone.0081859.s005.tif]

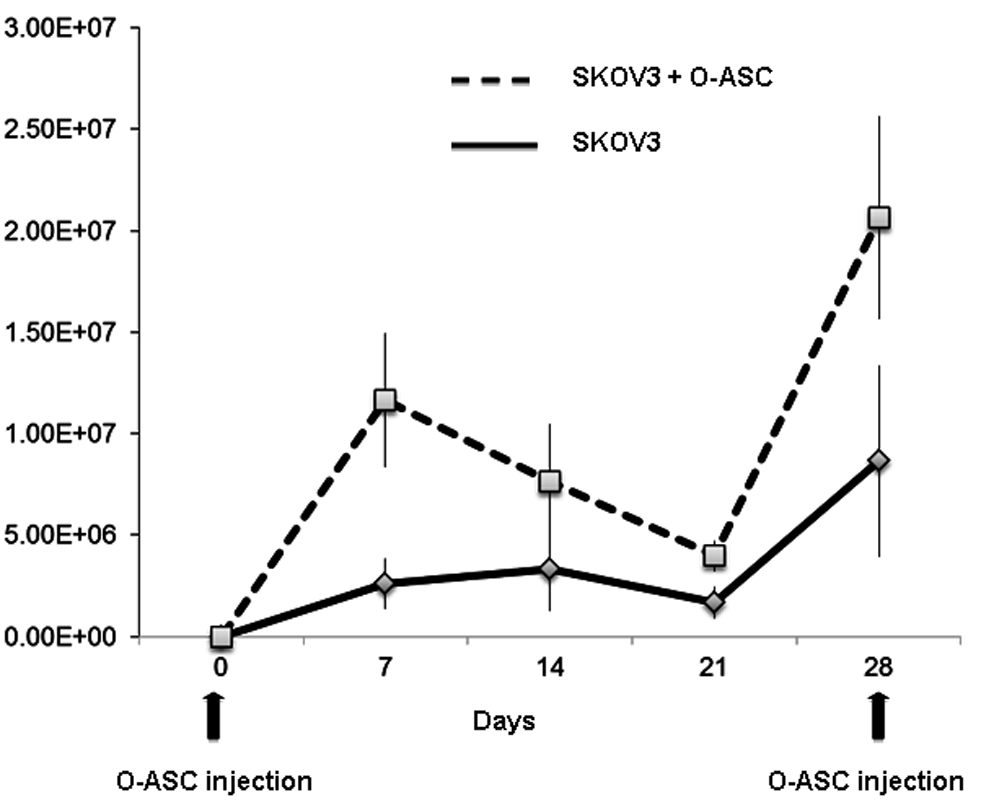

Supplement: Figure S6 — Invivo growth of SKOV3 tumors with and without O-ASC. Nude mice were injected with 5 x 10^6^ luciferase expressing SKOV3 cells (n = 4) with and without the same number of O-ASC (n = 5). Tumor growth was monitored with bioluminescent imaging. (TIF) [file pone.0081859.s006.tif]
